# Die Bedeutung von Lotsendiensten in Geburtskliniken für die weiterführende Versorgung von Familien

**DOI:** 10.1007/s00103-026-04304-9

**Published:** 2026-09-09

**Authors:** Maria Hänelt, Petra Steffen, Ilona Renner

**Affiliations:** 1 Nationales Zentrum Frühe Hilfen (NZFH) im Bundesinstitut für öffentliche Gesundheit (BIÖG), Maarweg 149–161, 50825 Köln, NRW Deutschland; 2 Deutsches Krankenhausinstitut e. V. (DKI), Düsseldorf, NRW Deutschland Prinzenallee 13 , 40549

**Keywords:** Patientensteuerung, Psychosoziale Belastungen, Bedarfsermittlung nach der Geburt, Unterstützungsangebote für Familien, Frühe Hilfen, Patient management, Psychological difficulties, Postnatal needs assessment, Support services for families, Early Childhood Intervention

## Abstract

**Hintergrund:**

Der Anteil psychosozial belasteter Familien auf den geburtshilflichen Stationen der Kliniken hat zugenommen. Deshalb ist eine kontinuierliche, evidenzbasierte Weiterentwicklung des Versorgungssystems dringend erforderlich. Untersucht werden soll, ob Lotsendienste in Geburtskliniken dazu beitragen können, die Patientensteuerung zu verbessern, indem Familien mit Unterstützungsbedarf aus der Geburtshilfe in passende psychosoziale und medizinische Angebote vermittelt werden.

**Methoden:**

Im Zeitraum 16.05. bis 31.08.2024 wurde allen 536 deutschen Geburtskliniken mit mehr als 300 Geburten pro Jahr ein Fragebogen zugesendet. Der Rücklauf betrug 48 % (256 Kliniken). Die Stichprobe ist bundesweit repräsentativ.

**Ergebnisse:**

Lotsendienste waren im Jahr 2024 in 67 % der Geburtskliniken in Deutschland eingerichtet. Kliniken mit Lotsendienst geben signifikant häufiger an, den Unterstützungsbedarf der (werdenden) Mütter und Familien systematisch zu ermitteln, als Kliniken ohne Lotsendienst. Häuser mit Lotsendienst leiten belastete Familien häufiger in weiterführende psychosoziale Angebote (Angebote, welche sich an einem spezifischen, festgestellten Unterstützungsbedarf orientieren) über.

**Diskussion:**

Belastungen von Familien können auf Geburtsstationen erkannt und näher exploriert werden. Lotsendienste in Geburtskliniken können die Versorgung der Familien optimieren und somit einzelne Elemente der Patientensteuerung für (werdende) Mütter erfüllen.

## Abstract (en)

**Background:**

The proportion of families identified as experiencing psychosocial difficulties in maternity clinics has increased. Therefore, evidence-based development of the healthcare system is urgently needed. This study aims to investigate whether family navigators in maternity clinics can contribute to improving patient management, specifically by referring families in need of support from the clinic to appropriate psychosocial and medical services.

**Methods:**

Between May 16 and August 31, 2024, a nationwide survey of all maternity clinics with more than 300 births per year was conducted. The response rate was 48% (256 clinics). The sample is representative nationwide.

**Results:**

In 2024, family navigators were place at 67% of maternity clinics in Germany. Clinics with family navigators reported significantly more often that they systematically assess the support needs of (expectant) mothers and families than clinics without such services. Facilities with family navigators more frequently refer families experiencing stress to further psychosocial services (services tailored to a specific, identified need for support).

**Discussion:**

Psychosocial difficulties experienced by families can be identified and explored in maternity clinics. Family navigators in maternity clinics can optimize care for families and thus fulfill specific aspects of patient management for (expectant) mothers.

## Einleitung

Die Patientensteuerung bezeichnet eine gezielte Lenkung von Patientinnen und Patienten durch das Gesundheitssystem, um sie zur richtigen Zeit zur richtigen Versorgungsstufe zu führen und gleichzeitig Überlastungen im Gesundheitssystem zu vermeiden [1, 2].

In diesem Beitrag soll der Frage nachgegangen werden, ob Gebärende in Geburtskliniken von Elementen einer (Patienten‑)Steuerung profitieren können. Zwar sind (werdende) Mütter im klassischen Sinne keine Patientinnen, da es sich bei der Geburt um einen natürlichen, physiologischen Prozess handelt. Dennoch werden sie in der Klinik formell als „Patientinnen“ geführt, da sie während und nach der Geburt bei Bedarf medizinische Versorgung und Angebote in Anspruch nehmen können.

In Deutschland wurden in Kliniken mit geburtshilflichen Stationen („Geburtskliniken“) verschiedene Formen der Patientensteuerung eingerichtet. Diese kann sowohl vor als auch nach der Geburt erfolgen. Vor der Geburt (Pränatalphase) umfasst sie die Koordination zwischen ambulanter Versorgung (Frauenärztinnen und -ärzte, Hebammen) und stationärer Geburt. Sie hat dann das Ziel einer bestmöglichen Versorgung der Schwangeren und des ungeborenen Kindes. Eine Steuerung nach der Geburt (Postnatalphase) umfasst die strukturierte Überleitung von der stationären Klinikversorgung in die ambulante Nachsorge durch Hebammen, Fachärztinnen und -ärzte sowie andere Unterstützungsangebote mit dem Ziel einer lückenlosen Versorgung von Mutter und Kind. Dabei kann die Patientensteuerung in der Geburtsklinik, bei Bedarf, über eine Vermittlung medizinischer Leistungen hinausgehen: Der Übergang zu psychosozialen Unterstützungsangeboten (zum Beispiel Angeboten der Frühen Hilfen [3]) stellt inzwischen einen weiteren, elementaren Bestandteil der Patientensteuerung nach der Geburt dar.

Ein Konzept der Patientensteuerung, das spezifisch auf die Situation psychosozial belasteter Frauen, die zur Geburt in eine Klinik kommen, zugeschnitten ist, erlangte in den letzten Jahren eine zunehmende Bedeutung: die Lotsendienste.

Lotsendienste sind auch in anderen Bereichen des Gesundheitssystems etabliert (z. B. Schlaganfall-Lotsen, Onkolotsen, Lotsen für an Demenz erkrankte Patientinnen und Patienten). Lotsendienste können als navigierende Schnittstelle im Gesundheitssystem fungieren [4]. Sie können Familien gezielt durch die verschiedenen Versorgungsstufen (z. B. von der Klinik in die ambulante Weiterversorgung) leiten und so die Belastung von Betroffenen senken [5]. Sie können dazu beitragen, Fehlversorgung zu vermeiden, Ressourcen gezielt einzusetzen und Versorgungslücken zu schließen [6].

Für die Umsetzung von Lotsendiensten in Geburtskliniken gibt es verschiedene Programme oder Konzepte sowie Standards. Hierzu zählt, dass Lotsendienste durch speziell ausgebildete Fachkräfte umgesetzt werden und konzeptuell abgesichert sind. Die Lotsinnen und Lotsen in Geburtskliniken sollen psychosoziale Belastungen der Mütter und Familien wahrnehmen und einordnen, ihnen noch in der Geburtsklinik Information und Beratung anbieten sowie sie bei Bedarf in örtlich vorhandene Unterstützungsangebote vermitteln [7]. Ein Lotsendienst sollte nach Schmenger et al. folgende Qualitätskriterien erfüllen: (a) Einschätzung des Hilfebedarfs, (b) vertiefende Beratung, (c) ggf. Überleitung und (d) fallübergreifende Teilnahme am lokalen Netzwerk (Frühe Hilfen; [8]).

Lotsendienste wurden in den letzten Jahren in immer mehr Geburtskliniken eingerichtet. Ein Vorteil der Tätigkeit von Lotsendiensten liegt in der Art der Steuerung: Sie erfolgt freiwillig und ist typischerweise niedrigschwellig und selbstbestimmt. Damit entspricht sie den Prämissen einer selbstbestimmten Patientensteuerung, welche den einzelnen Patientinnen und Patienten die Fähigkeit zur Einwilligung sowie die Eigenverantwortlichkeit bei Entscheidungen über medizinische Behandlungen zuspricht [9]. Im Fall der Lotsendienste geht die Eigenverantwortlichkeit über das medizinische System hinaus und umfasst die potenzielle Überleitung in Unterstützungsangebote zur Reduzierung von psychosozialen Belastungen. Dies setzt voraus, dass die individuellen Bedürfnisse und Werte der (werdenden) Mütter in Gesprächen systematischer ermittelt werden, um passgenaue Unterstützung anbieten zu können. Die systematische Ermittlung erfolgt in den Geburtskliniken vor allem durch Einsatz von Screening-Instrumenten oder Einschätzungsbögen, durch (dokumentierte) Einschätzung des Stationspersonals nach Gesprächen mit den Eltern oder durch (dokumentierte) Lotsenbesuche der Eltern vor und/oder nach der Geburt eines Kindes [6].

Bisher unbeantwortet bleibt die Frage, inwieweit der Einsatz eines Lotsendienstes in Geburtskliniken als Patientensteuerung verstanden wird und inwieweit er zu einer besseren Versorgung der (werdenden) Mütter betragen kann. Deshalb soll im vorliegenden Beitrag der Frage nachgegangen werden, *inwieweit der Lotsendienst in Geburtskliniken die Funktion einer Patientensteuerung übernimmt und dazu beitragen kann, dass Familien mit Unterstützungsbedarf in weiterführende Angebote vermittelt werden.*

Grundvoraussetzung dafür, dass Patientensteuerung gelingt bzw. dass Familien mit Hilfebedarf in passende Angebote übergeleitet werden, ist, dass familiale Belastungen in den Kliniken grundsätzlich feststellbar sind. Dies ist jedoch in Anbetracht der aktuellen Rahmenbedingungen im Klinikalltag, wie etwa der hohen Arbeitsbelastung, der immer kürzeren Liegezeiten (werdender) Mütter und des Fachkräftemangels, erschwert [10]. Bei Erfüllung dieser Grundvoraussetzung ist eine systematische Ermittlung des Unterstützungsbedarfs notwendig, um die eigentlichen Belastungen sowie passgenaue Unterstützungsangebote identifizieren zu können [8]. Die Implementierung eines eigens dafür eingerichteten Lotsendienstes könnte durch die konzeptuelle Einbettung im Klinikalltag dazu beitragen, dass dies auch unter den skizzierten Rahmenbedingungen gelingt. Deshalb sollte zunächst geprüft werden, inwieweit ein Lotsendienst zu einer systematischeren Ermittlung des Unterstützungsbedarfs beitragen kann.

*Hypothese I: Kliniken mit Lotsendienst ermitteln Belastungen von Familien häufiger systematisch als Kliniken ohne Lotsendienst.*

Ein relevantes Qualitätskriterium für Lotsendienste in Geburtskliniken ist die Einbindung in das lokale Netzwerk. Lotsendienste können sich dadurch neben der Kenntnis klinikinterner Angebote auch eine Übersicht zu klinikexternen Angeboten aneignen und so eine größere Bandbreite von passgenaueren Vermittlungsoptionen für (werdende) Eltern anbieten. Deshalb soll überprüft werden, inwieweit Kliniken mit Lotsendienst über eine umfangreichere Übersicht über die kommunale Unterstützungslandschaft verfügen.

*Hypothese II: Kliniken mit Lotsendienst geben mehr Vermittlungsoptionen für belastete Familien an als Kliniken ohne Lotsendienst.*

Es erscheint plausibel, dass sich hierdurch die tatsächliche Weiterleitung in (interne und externe) Unterstützungsangebote erhöht. Falls, wie oben hypothetisiert, Kliniken mit Lotsendienst mehr Kenntnisse über Unterstützungsangebote vor Ort haben als Kliniken ohne Lotsendienst, ist davon auszugehen, dass sie auch häufiger in weiterführende psychosoziale Angebote vermitteln. Unter „weiterführenden psychosozialen“ Angeboten sollen hier Angebote verstanden werden, die sich an einem spezifischen, festgestellten Unterstützungsbedarf orientieren und nicht wieder in das medizinische System (also zu Ärztinnen und Ärzten oder Therapeutinnen und Therapeuten) zurückvermitteln.

Das Ziel ist es, Angebote zu machen, die die spezifischen Ursachen der Belastung betreffen, somit eine Fehlversorgung vermeiden und gleichzeitig das „klassische“ Gesundheitssystem entlasten. Beispiele hierfür können die Suchtberatung – für Eltern mit Suchterkrankungen – oder Migrationsdienste – für Eltern mit Verständigungsschwierigkeiten – sein. Dabei muss darauf hingewiesen werden, dass nicht jede Belastung durch ein passendes Unterstützungsangebot zu versorgen ist und nicht jede Familie trotz Identifikation von Belastungen die Vermittlung in ein spezifisches Angebot wünscht. Auch eine Weiterleitung in das medizinische System kann zielführend sein oder dem Wunsch der Familie entsprechen. Lotsendienste richten sich als präventives Angebot an Familien, wobei die Inanspruchnahme stets auf freiwilliger Basis erfolgt.

*Hypothese III: Kliniken mit Lotsendienst vermitteln Familien eher in weiterführende psychosoziale Angebote als Kliniken ohne Lotsendienst.*

## Methoden

Das Nationale Zentrum Frühe Hilfen (NZFH) hat das Deutsche Krankenhausinstitut e. V. (DKI) damit beauftragt, eine bundesweite Vollerhebung unter allen Geburtskliniken mit mehr als 300 Geburten pro Jahr durchzuführen. Das NZFH wird vom Bundesministerium für Familie, Senioren, Frauen und Jugend gefördert. Die Träger sind das Bundesinstitut für Öffentliche Gesundheit (BIÖG) und das Deutsche Jugendinstitut (DJI).

Die Datenerhebung fand im Zeitraum vom 16.05. bis zum 31.08.2024 in 3 Wellen statt. Alle Kliniken mit geburtshilflichen Fachabteilungen mit mehr als 300 Geburten pro Jahr erhielten einen Fragebogen, der von einer oder mehreren Personen unterschiedlicher Berufsgruppen ausgefüllt werden konnte. Das Klinikpersonal hatte die Möglichkeit, den Fragebogen entweder schriftlich oder online zu bearbeiten. Insgesamt wurden 536 Kliniken angeschrieben, wovon 256 Kliniken an der Befragung teilnahmen. Das entspricht einem Rücklauf von 48 %. Um systematische Verzerrungen (Non-Response-Bias) zu ermitteln, wurde eine Non-Responder-Befragung unter den Kliniken durchgeführt, die nicht an der Befragung teilgenommen haben. Hier wurde unter anderem ermittelt, ob die Kliniken einen Lotsendienst bereits etabliert haben oder dieser in der Planung ist.

### Instrument

Der Fragebogen wurde auf Grundlage einer im Jahr 2017 durchgeführten Erhebung in Geburtskliniken weiterentwickelt. Dabei wurden Erkenntnisse aus aktuellen Studien sowie Anregungen aus Fachkräfteinterviews und Pretests berücksichtigt. Der Fragebogen umfasste mehrere Abschnitte, darunter die Erhebung allgemeiner Informationen zur Klinik, Informationen zur Implementierung von Lotsendiensten, eine Einschätzung der Belastungslagen der (werdenden) Mütter und ihrer Familien sowie Informationen zu konkreten Maßnahmen zur psychosozialen Versorgung belasteter Familien auf der Geburtsstation und deren Weiterleitung in interne und externe Versorgungsangebote.

#### Identifikation Lotsendienst.

Die Fachkräfte wurden nach der Umsetzung des Lotsendienstes in ihrer Geburtsklink befragt. Dazu wurde im Fragebogen ein „Lotsendienst“ zunächst wie folgt definiert: „Bei einem Lotsendienst bieten Fachkräfte (psychosozial) belasteten Familien niedrigschwellig Information und Beratung an und leiten sie bei Bedarf weiter. Die Fachkräfte sprechen Familien systematisch an, schätzen gemeinsam mit den Eltern etwaigen Unterstützungsbedarf ein und bringen sie – bei Bedarf – mit weitergehenden Unterstützungsangeboten in Kontakt“ (Definition des NZFH). Auf die Definition folgte die Frage: *Haben Sie einen Lotsendienst für belastete (werdende) Familien (z.B. Familienlotsin/-lotse, Babylotsin/-lotse, Guter Start ins Kinderleben) in Ihrer Klinik eingerichtet? *Es handelt sich hierbei also um eine Einschätzung des befragten Klinikpersonals. Die Teilnehmenden der Befragung konnten unter folgenden Antwortoptionen wählen: (a) „Ja“, (b) „Nein, aber konkret in Planung“, (c) „Nein, aber grundsätzliches Interesse“, (d) „Nein, aber hatten wir früher mal“ und (e) „Nein, und kein Interesse“.

#### Systematik in der Ermittlung der Belastungen.

Auch bei der Frage nach der systematischen Ermittlung des Unterstützungsbedarfs der Familien handelt es sich um eine Einschätzung der Teilnehmenden an der Befragung. Die Frage lautete: *Wird der (psychosoziale) Unterstützungsbedarf bei (werdenden) Eltern systematisch ermittelt?* Es wurde dichotomisiert abgefragt (Ja/Nein).

#### Vermittlungsoptionen (intern und extern).

Die Teilnehmenden der Befragung wurden nach Angeboten gefragt, die in der jeweiligen Klinik (intern) sowie im Einzugsgebiet der Klinik (extern) für (werdende) Eltern zur Verfügung stehen. *Welche Angebote hält Ihre Klinik intern für (werdende) Eltern vor? *und *Welche Angebote außerhalb Ihrer Klinik sind im Einzugsgebiet der Klinik für (werdende) Eltern erreichbar?* Es wurden 16 interne und 24 externe Angebote abgefragt. Auch „sonstige“ Angebote konnten angegeben werden. Mehrfachantworten waren möglich.

#### Vermittlung in Angebote (intern und extern).

Die Teilnehmenden wurden dazu befragt, ob sie belastete Familien in verschiedene Angebote vermitteln. *Vermitteln Sie belastete Familien regelmäßig in folgende Angebote?* Antwortoptionen: „Ja, vermittle“; „Nein, da keine/zu wenig Plätze“ und „Nein, vermittele nicht, weil kein Bedarf“. Es wurden 24 verschiedene klinikinterne und klinikexterne sowie weiterführende psychosoziale und medizinische Angebote abgefragt (Abb. 2 im Ergebnisteil). Medizinische Angebote umfassen dabei die psychotherapeutischen Praxen, die gynäkologischen Praxen und die pädiatrischen Praxen. Alle anderen abgefragten Angebote werden als weiterführende psychosoziale Angebote verstanden.

## Ergebnisse

Die Stichprobe ist repräsentativ für die Grundgesamtheit der Geburtskliniken in Deutschland. Es zeigen sich keine signifikanten Unterschiede zwischen Grundgesamtheit und Stichprobe in den relevanten Kriterien, wie z. B. Bettenanzahl oder Trägerschaft (ZuFa[Zusammen für Familien]-Monitoring 2024/Nutricia-Geburtenliste 2023; [11]; Tab. 1).

**Tab. 1:** Vergleich von Strukturdaten aller Geburtskliniken (teilnehmende und nichtteilnehmende) mit mehr als 300 Geburten pro Jahr. (Datenquelle: Nutricia-Geburtenliste 2023 [11]).

Kriterien	Grundgesamtheit: Anteil bei Kliniken insgesamt (*N* = 536)	Stichprobe: Anteil bei teilnehmenden Kliniken (*n* = 256)
*Größe der Klinik: Bettenzahl >600*	22 %	24 %
*Kliniken in kreisfreien Großstädten*	33 %	40 %
*Kliniken in städtischen Kreisen*	36 %	32 %
*Kliniken in ländlichen Kreisen mit Verdichtungsansätzen*	18 %	17 %
*Kliniken in dünn besiedelten ländlichen Kreisen*	13 %	12 %
*Öffentliche Trägerschaft*	46 %	44 %

67 % aller befragten Kliniken mit geburtshilflichen Fachabteilungen (*N* = 256) gaben an, einen Lotsendienst für belastete (werdende) Familien eingerichtet zu haben oder diesen konkret zu planen. Die Non-Responder-Befragung konnte zeigen, dass keine starken Verzerrungen bezüglich der Etablierung von Lotsendiensten in den Kliniken bestehen, die an der Befragung teilgenommen haben. Von den Kliniken, die nicht an der Befragung teilnahmen, gaben 60 % an, dass ein Lotsendienst umgesetzt ist oder konkret in Planung ist.

Es zeigen sich Unterschiede nach siedlungsstrukturellen Kreistypen: Die teilnehmenden Kliniken in kreisfreien Großstädten (47 %, Tab. 2) und städtischen Kreisen (31 %, Tab. 2) haben signifikant häufiger einen Lotsendient implementiert als Kliniken in ländlichen Regionen (14 % bzw. 9 %, Tab. 2). Kliniken mit Lotsendienst hatten im Jahr 2023 signifikant höhere Geburtszahlen (Mittelwert = 1383 vs. 1006; Tab. 2), wobei die Geburtenzahlen signifikant mit den siedlungsstrukturellen Kreistypen zusammenhängen. Die Geburtenzahlen liegen in Kliniken in (Groß‑)Städten über denen in ländlichen Regionen.

**Tab. 2:** Vergleich von Strukturdaten der teilnehmenden Kliniken mit und ohne Lotsendienst

Kriterien	Anteil bei Kliniken mit Lotsendienst (*n* = 165)	Anteil bei Kliniken ohne Lotsendienst (*n* = 81)
*Größe der Klinik: Bettenzahl >600*	26 %	20 %
*Kliniken in kreisfreien Großstädten*	47 %	25 %
*Kliniken in städtischen Kreisen*	31 %	33 %
*Kliniken in ländlichen Kreisen mit Verdichtungsansätzen*	14 %	24 %
*Kliniken in dünn besiedelten ländlichen Kreisen*	9 %	19 %
*Öffentliche Trägerschaft*	40 %	54 %
*Geburtenzahl pro Jahr (Durchschnittswerte)*	1383 (SD: 794)	1006 (SD: 658)

*SD* Standardabweichung

Für eine Mehrheit der teilnehmenden Kliniken, egal ob mit oder ohne Lotsendienst, gilt, dass sich die Befragten grundsätzlich dazu in der Lage sehen, im beruflichen Alltag psychosoziale Belastungen von Familien festzustellen (Details siehe Faktenblatt [12]). Die Einschätzung, dass die Geburtshilfe grundsätzlich ein Setting ist, das sich zur Identifikation psychosozialer Belastungen bei den (werdenden) Müttern und ihren Familien eignet, ist unabhängig davon, ob Kliniken einen Lotsendienst eingerichtet haben oder nicht. Kliniken mit und ohne Lotsendienste unterscheiden sich hier nicht signifikant voneinander.

**Abb. 1:**
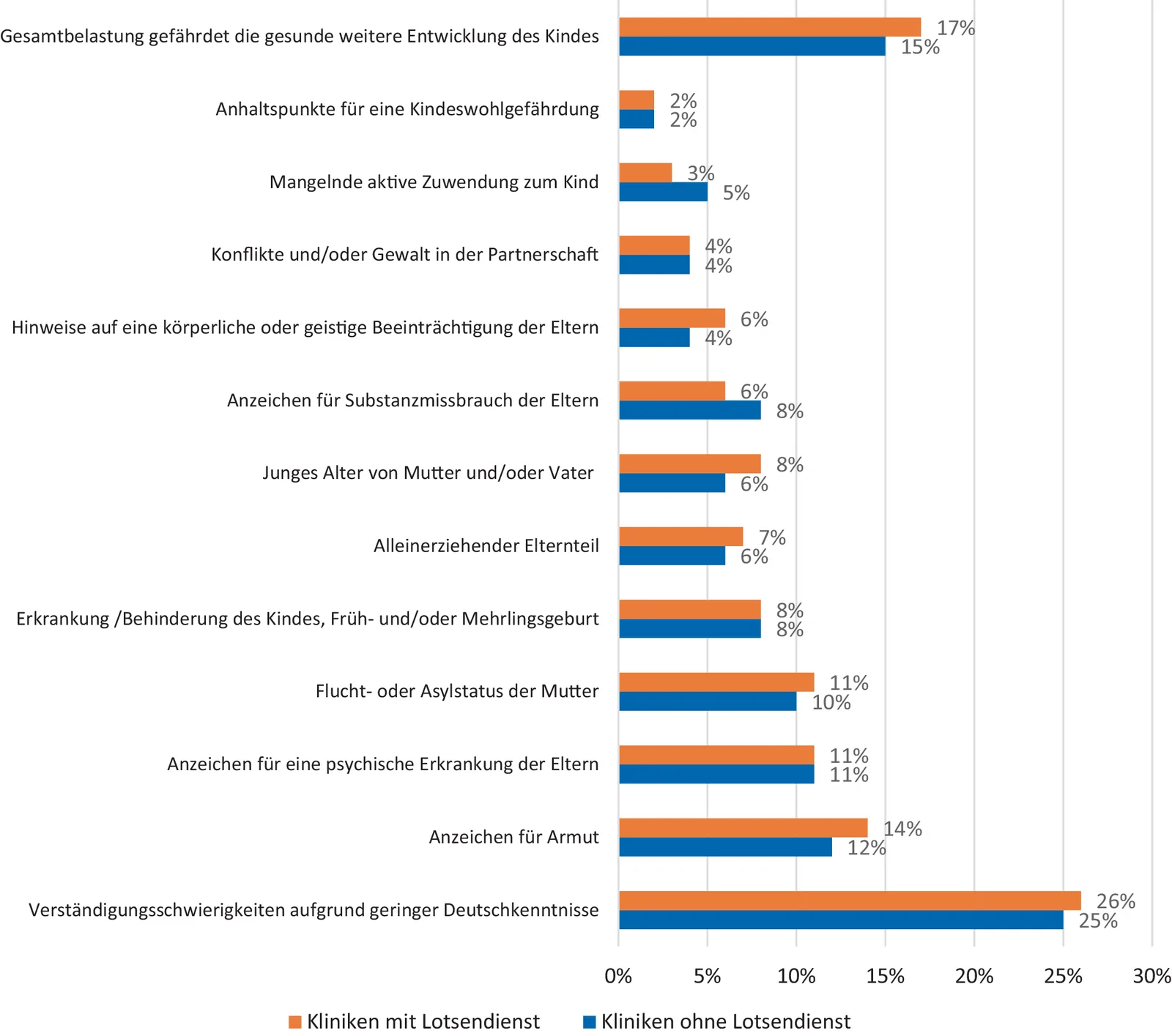
Anteil belasteter Familien nach unterschiedlichen Belastungsfaktoren (Frage: „Bei wie vielen dieser 100 Eltern oder Kinder machen Sie folgende Beobachtungen?“); *n* = 256

Auch die Höhe des Anteils von Familien in Belastungslagen (Abb. 1) ist unabhängig davon, ob ein Lotsendienst eingerichtet ist oder nicht. Zwischen Kliniken mit und ohne Lotsendienst gibt es keine signifikanten und systematischen Unterschiede im Belastungsniveau der (werdenden) Mütter und ihrer Familien. Klinikübergreifend nehmen die Häuser wahr, dass die psychosozialen Belastungen bei den versorgten Familien in den letzten Jahren zugenommen haben. Das betrifft vor allem „Verständigungsschwierigkeiten aufgrund unzureichender Deutschkenntnisse“ sowie die „Anzeichen für eine psychische Erkrankung der Eltern“.

### Systematische Ermittlung von belasteten Familien

*Hypothese I: Kliniken mit Lotsendienst ermitteln Belastungen von Familien häufiger systematisch als Kliniken ohne Lotsendienst.*

Für die gezielte Steuerung von (werdenden) Müttern ist es zentral, den möglicherweise aus familialen Belastungen resultierenden (psychosozialen) Unterstützungsbedarf bei (werdenden) Eltern systematisch zu ermitteln.

Etwas mehr als die Hälfte der teilnehmenden Kliniken (59 %) gibt an, Unterstützungsbedarf systematisch zu ermitteln. Hierbei zeigen sich signifikante Unterschiede zwischen Kliniken mit und ohne Lotsendienst. Kliniken mit Lotsendienst geben signifikant häufiger an, den Unterstützungsbedarf der Familien systematisch zu eruieren (71 %) als Kliniken ohne Lotsendienst (35 %).

### Vermittlungsoptionen für belastete Familien

*Hypothese II: Kliniken mit Lotsendienst geben mehr Vermittlungsoptionen für belastete Familien an als Kliniken ohne Lotsendienst.*

Die Kliniken wurden gebeten anzugeben, welche Angebote das Krankenhaus selbst für (werdende) Eltern vorhält (klinikintern) und welche Unterstützungsmöglichkeiten darüber hinaus im Einzugsgebiet der Klinik erreichbar sind (klinikextern). Es zeigen sich statistisch signifikante Unterschiede zwischen den Kliniken mit und ohne Lotsendienst. Kliniken mit Lotsendienst halten im Durchschnitt 8,5 verschiedene klinikinterne Angebote für (werdende) Eltern und Familien vor, während Kliniken ohne Lotsendienst 7,3 Angebote vorhalten. Eine ähnliche Tendenz zeigt sich bei den klinikexternen Angeboten: Kliniken mit Lotsendienst geben im Durchschnitt an, dass 21,3 klinikexterne Angebote im Einzugsgebiet der Klinik für werdende Eltern erreichbar sind, während für Kliniken ohne Lotsendienst 19,8 Angebote angegeben werden.

Mitarbeitende in Geburtskliniken mit Lotsendienst haben mehr weiterführende psychosoziale Angebote als Vermittlungsoption als Mitarbeitende in Geburtskliniken ohne Lotsendienst, beispielsweise: Elternkurse bzw. Familienbildungsstätten (81 % vs. 66 %); Familienpflegende bzw. Familienpatinnen bzw. -paten (70 % vs. 43 %); sozialpädiatrische Zentren (70 % vs. 51 %) und Migrationsdienste/-einrichtungen bzw. Erstaufnahmeeinrichtungen (77 % vs. 58 %).

### Vermittlung in weiterführende psychosoziale Angebote

*Hypothese III: Kliniken mit Lotsendienst vermitteln Familien eher in weiterführende psychosoziale Angebote als Kliniken ohne Lotsendienst.*

**Abb. 2:**
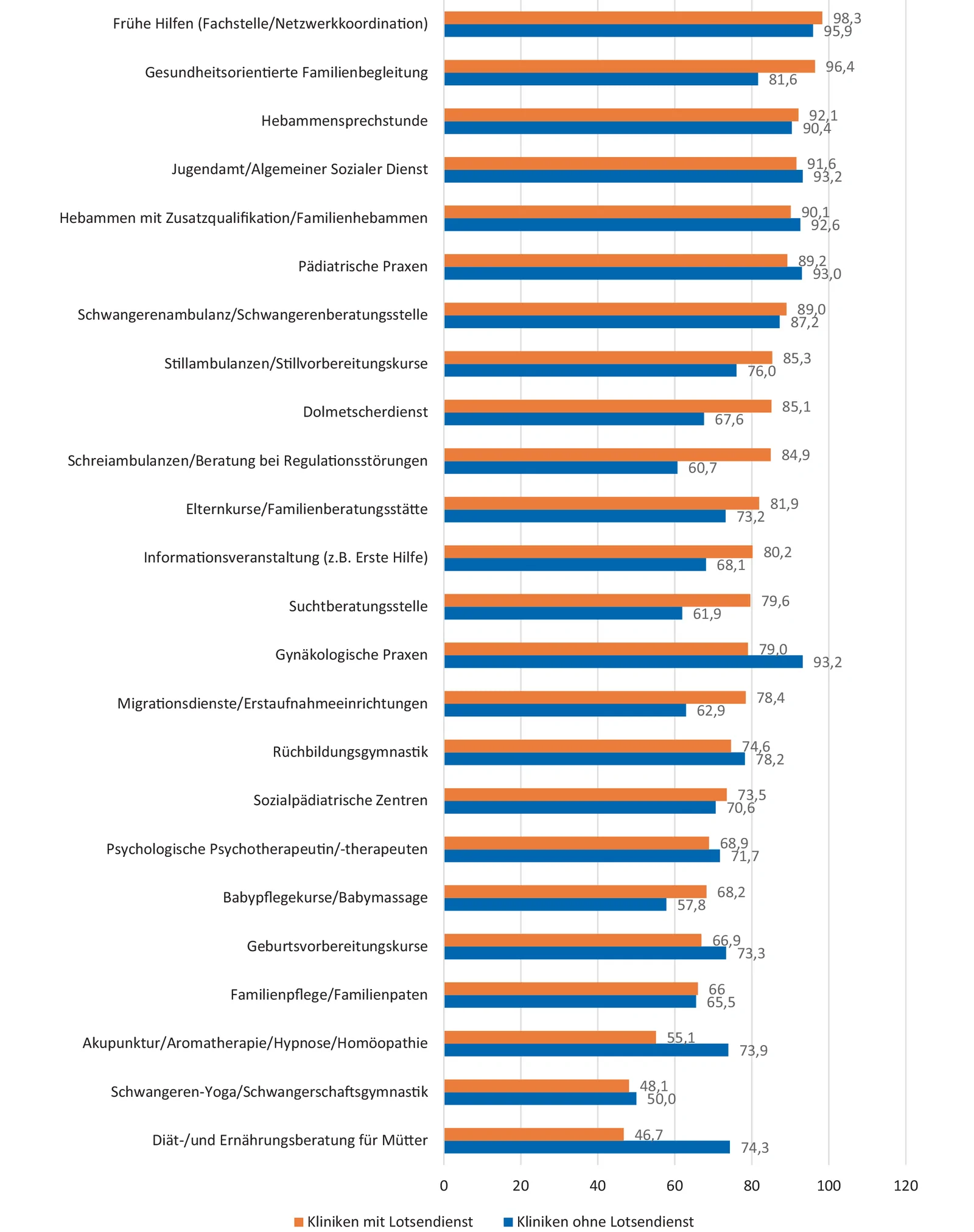
Anteile der Kliniken mit und ohne Lotsendienst, die regelmäßig in die aufgeführten Angebote vermitteln (Frage: „Vermitteln Sie belastete Familien regelmäßig in folgende Angebote?“; Antwort: Ja, ich vermittle); *n* = 256. * signifikanter Unterschied zwischen Kliniken mit Lotsendienst und Kliniken ohne Lotsendienst. Angaben in %

Bei der Vermittlung der belasteten Familien in Angebote zeigen sich signifikante Unterschiede zwischen Kliniken, die einen Lotsendienst eingerichtet haben, und Kliniken ohne Lotsendienst (Abb. 2). Häuser mit Lotsendienst leiten belastete Familien häufiger in die gesundheitsorientierte Familienbegleitung (Familien-Hebammen oder Familien-Gesundheits- und Kinderkrankenpflegerinnen und -pfleger; 96 % vs. 82 %) oder informieren über Angebote wie Schreiambulanzen (85 % vs. 61 %). Unterschiede in der Vermittlung zeigen sich tendenziell auch für andere weiterführende psychosoziale Angebote wie die Stillberatung (85 % vs. 73 %), den Dolmetscherdienst (85 % vs. 68 %) oder die Suchtberatungsstelle für Eltern (80 % vs. 62 %).

Dahingegen vermitteln Klinken mit Lotsendienst signifikant weniger als Kliniken ohne Lotsendienst in gynäkologische Praxen (79 % vs. 93 %) und Diät- bzw. Ernährungsangebote für Mütter (85 % vs. 47 %).

Nicht unerwähnt bleiben soll hier ein wichtiger Befund, der sowohl für Kliniken mit als auch für Kliniken ohne Lotsendienst gilt: Mitarbeitende der teilnehmenden Kliniken geben an, dass es Angebote gibt, für die ein Bedarf besteht, in welche aufgrund fehlender Plätze aber nicht entsprechend vermittelt werden kann. Zu diesen Angeboten zählen: psychologische Psychotherapeutinnen und Psychotherapeuten (26 %) und Dolmetscherdienste (15 %). Dies ist vor allem vor dem Hintergrund relevant, dass psychische Belastungen der Eltern und Verständigungsschwierigkeiten aufgrund geringer Deutschkenntnisse zu den Belastungen zählen, die laut Klinikpersonal am häufigsten auftreten (Details siehe Faktenblatt [12]).

## Diskussion

Die Ergebnisse der vorliegenden Studie liefern Einblicke in die Rolle von Lotsendiensten in der geburtshilflichen Versorgung. Psychosoziale Belastungen können grundsätzlich durch das Klinikpersonal festgestellt werden; die Grundvoraussetzung für eine Steuerungs- und Lotsenfunktion ist somit erfüllt. Hinsichtlich der Wahrnehmung psychosozialer Belastungen zeigt sich kein Unterschied zwischen Kliniken mit und ohne Lotsendienst. Dies macht deutlich, dass Belastungen bei (werdenden) Müttern und ihren Familien nicht allein deshalb häufiger wahrgenommen und ggf. überschätzt werden, weil in der Klinik ein Lotsendienst vorhanden ist. Insgesamt geben die Kliniken an, dass die Belastungen der Familien zugenommen haben. Ein Vergleich der Daten aus 2017 und 2024 zeigt, dass laut Klinikpersonal (aus Kliniken mit und ohne Lotsendienst) vor allem die „Verständigungsschwierigkeiten aufgrund unzureichender Deutschkenntnisse“ sowie die „Anzeichen für eine psychische Erkrankung der Eltern“ in den letzten Jahren an Bedeutung gewonnen haben [12].

Obwohl die Mehrheit der befragten Kliniken den Unterstützungsbedarf (werdender) Familien rund um die Geburt erhebt, zeigt sich ein signifikanter Unterschied zwischen Einrichtungen mit und ohne Lotsendienst. Kliniken mit Lotsendienst erfassen den Unterstützungsbedarf signifikant häufiger systematisch als Kliniken ohne Lotsendienst. Ein systematisches Vorgehen kann nicht nur zu einer gezielteren Erfassung der Belastungen, sondern auch zu zielgerichteteren Handlungsempfehlungen beitragen, da es eine vertiefende Beratung und ggf. eine zielgenaue Überleitung ermöglicht. Da es sich bei der Befragung um eine Selbsteinschätzung des Klinikpersonals handelt, kann allerdings nicht abschließend geklärt werden, was die Teilnehmenden der Studie unter einem „systematischen“ Vorgehen verstehen. Es ist davon auszugehen, dass durch die Implementierung von Lotsendiensten, die häufig mit einer schriftlichen Definition verbindlicher Ziele einhergeht, die Grundlagen für eine systematische Arbeitsweise gelegt werden.

In den befragten Kliniken mit Lotsendiensten sind mehr interne und externe Angebote für (werdende) Eltern angesiedelt bzw. werden als „erreichbar“ eingeschätzt als in Kliniken ohne Lotsendienst. Es ist zu vermuten, dass Lotsendienste einen besseren Überblick über die (interne und vor allem externe) Angebotslandschaft aufweisen, weil sie sowohl innerhalb als auch außerhalb der Klinik besser vernetzt sind [13]. Dies erleichtert eine bedarfsorientiertere Weiterleitung der Familien in Angebote. Die Ergebnisse liefern Hinweise darauf, dass eine bedarfsorientierte Vermittlung in Kliniken mit Lotsendiensten eher umgesetzt wird als in Kliniken ohne Lotsendienst. So vermitteln Kliniken mit Lotsendienst häufiger als Kliniken ohne Lotsendienst in weiterführende psychosoziale Unterstützungsangebote wie Familienhebammen. Kliniken ohne Lotsendienst vermitteln hingegen häufiger als Kliniken mit Lotsendienst in regelhafte Angebote wie gynäkologische Praxen.

Diese Befunde legen nahe, dass Lotsendienste eine koordinierende Funktion (innerhalb und außerhalb der Klinik) erfüllen können und zu einer Weiterleitung in externe Hilfesysteme beitragen. Auch wenn die Frage, inwieweit der Einsatz eines Lotsendienstes in Geburtskliniken als Patientensteuerung verstanden werden kann, hier nicht abschließend beantwortet werden kann, enthält die Ausgestaltung der Lotsendienste in den Geburtskliniken Kernelemente einer Patientensteuerung. So tragen sie offenbar dazu bei, Familien systematisch in weiterführende Angebote zu vermitteln.

Lotsendienste können auf diesem Weg zu einer niedrigschwelligen und selbstbestimmten Patientensteuerung beitragen und die Eigenverantwortlichkeit der Familien bei der Vermittlung in weiterführende Angebote fördern. Dabei ist es wichtig, zu erwähnen, dass auch eine Übermittlung in das medizinische System eine notwendige und fachgemäße Entscheidung sein kann und nicht jede Belastung ist durch ein passendes psychosoziales weiterführendes Unterstützungsangebot zu versorgen.

Klinikübergreifend berichten die Fachkräfte auch von Belastungen der Eltern, die aufgrund fehlender Versorgungsangebote nicht vermittelt werden können. Dazu gehören vor allem psychologische Psychotherapeutinnen und Psychotherapeuten und Dolmetscherdienste. Dies ist vor allem vor dem Hintergrund relevant, dass psychische Belastungen der Familien und Verständigungsschwierigkeiten mit Fachkräften zu den Belastungen von Familien zählen, die in den letzten Jahren zugenommen haben [12]. Patientensteuerung kann nur erfolgreich sein, wenn für eine identifizierte Belastung ein entsprechendes Unterstützungsangebot zur Verfügung steht.

### Limitationen und weiterführende Forschung

Noch nicht beantwortet ist die Frage, ob jene Familien, die vom Klinikpersonal in ein Angebot vermittelt wurden, dieses auch nutzen beziehungsweise dieses in Anspruch nehmen. Erst dann kann die Frage geklärt werden, ob eine Patientensteuerung mittels Lotsendienstes erfolgreich ist. Zukünftige Studien sollten daher zum einen gezielt die Perspektive der Eltern einbeziehen und untersuchen, wie erfolgreiche Patientensteuerung aus Sicht der Familien definiert und erlebt wird. Zum anderen sollten Überleitungserfolg und Inanspruchnahme der entsprechenden Angebote untersucht werden. Auch zur Frage, ob die Vermittlung bedarfsgerecht ist, kann die vorliegende Studie nur erste Hinweise liefern. Weiterführende Forschung sollte Daten zu Belastungen und Bedarfen und Daten zu Identifizierung und Vermittlung fallbezogen zusammenführen.

Anhand der Studienergebnisse lässt sich nicht beurteilen, ob die Etablierung eines Lotsendienstes die Angebotsstruktur beeinflusst oder umgekehrt. Ebenso kann nicht beurteilt werden, ob die Geburtenzahlen sich im Zuge der Etablierung des Lotsendienstes in der Klinik verändert haben. Diese Studie betrachtet die Ist-Situation. Folgestudien sind notwendig, die mithilfe größerer Fallzahlen weiterführende Zusammenhangsanalysen durchführen können.

Darüber hinaus basieren die erhobenen Daten ausschließlich auf Einschätzungen des Fachpersonals stellvertretend für die Kliniken, was potenzielle Verzerrungen implizieren kann. Auch der Effekt der sozialen Erwünschtheit bezüglich des Antwortverhaltens der Fachkräfte kann zu Verzerrungen führen. Vor der Selbsteinschätzung wurde die Tätigkeit des Lotsendienstes im Fragebogen definiert. Zu der Definition der Tätigkeit gehörte auch das systematische Vorgehen von Lotsen. Die Kenntnis über die Definition kann die Fachkräfte dazu veranlasst haben, von einem systematischen Vorgehen in der eigenen Klinik zu berichten.

## Fazit

Vor dem Hintergrund zunehmender Belastungen von Familien [12] ist eine bedarfsgerechte Vermittlung innerhalb und außerhalb des medizinischen Systems rund um die Geburt wichtig. Die vorliegenden Befunde weisen darauf hin, dass psychosoziale Belastungen auf Geburtsstationen grundsätzlich erkannt und weitergehend exploriert werden können. Lotsendienste können hierbei zu einer systematischeren Identifikation von Belastungslagen und einer zielgerichteten Vermittlung in geeignete Unterstützungsangebote beitragen. Kliniken mit implementiertem Lotsendienst weisen eine höhere Überleitungsquote in weiterführende psychosoziale Angebote auf als Einrichtungen ohne Lotsendienst. Die Studie erlaubt jedoch keine Aussagen darüber, ob diese Angebote tatsächlich in Anspruch genommen werden und ob die Vermittlung in die jeweils passenden spezifischen Angebote erfolgt. Insgesamt weisen die Ergebnisse darauf hin, dass Lotsendienste durch eine systematische Identifikation von Unterstützungsbedarfen und eine bedarfsgerechte Überleitung zur Optimierung der Versorgung beitragen können. In einem weiteren Evaluationsschritt sollten verstärkt patientenrelevante Endpunkte berücksichtigt und kausale Wirkungspfade untersucht werden.

## Data Availability

Der in der vorliegenden Studie erzeugte und analysierte Datensatz ist aus Gründen der Anonymitätswahrung nicht öffentlich zugänglich, kann aber auf begründete Anfrage bei den entsprechenden Autorinnen angefordert werden.
